# Evaluation of occupational stress and job performance in Iranian nurses: the mediating effect of moral and emotional intelligence

**DOI:** 10.1186/s12888-023-05277-8

**Published:** 2023-10-21

**Authors:** Vahid Alinejad, Naser Parizad, Laleh Almasi, Rozita Cheraghi, Mehri Piran

**Affiliations:** 1 Department of Biostatistics, School of Medicine, Urmia University of Medical Sciences, Urmia, Iran; 2 Patient Safety Research Center, Clinical Research Institute, School of Nursing & Midwifery, Urmia University of Medical Sciences, Urmia, Iran; 3 Department of Medical-Surgical Nursing, School of Nursing & Midwifery, Urmia University of Medical Sciences, Urmia, Iran; 4 Imam Khomeini Hospital, Urmia University of Medical Sciences, Urmia, Urmia, Iran

**Keywords:** Nurses, Occupational stress, Job performance, Emotional intelligence, Moral, Iran

## Abstract

**Background:**

Nurses’ Job performance could be affected by occupational stress. Previous studies reported contradictory results in this regard. Factors such as moral and emotional intelligence could impact occupational stress. However, the extent of any mediating effect is unclear. Thus, this study aimed to determine the effect of occupational stress on nurses’ Job performance and the mediating impact of moral and emotional intelligence.

**Methods:**

This cross-sectional study was conducted in Urmia teaching hospitals (Imam Khomeini, Motahari, Taleghani, Kosar, and Seyed al-Shohada Hospitals). Six hundred twenty-one nurses were selected using quota sampling from February 2022 to April 2022. Data were collected using demographic questionnaires, the Nursing Stress Scale, Paterson’s Job Performance Questionnaire, Siberia Schering’s Emotional Intelligence Standard Questionnaire, and Lennik and Keil’s Moral Intelligence Questionnaire. Data were analyzed using SPSS ver. 23 and SmartPLS ver. 2.

**Results:**

Occupational stress had a positive, direct, and minor effect on nurses’ job performance (β = 0.088, t-value = 2.245, *p* < 0.01). Occupational stress had a positive and direct impact on moral intelligence (β = 0.161, t-value = 2.945, *p* < 0.01) and a negative and direct effect on emotional intelligence (β = -0.351, t-value = 7.484, *p* < 0.01). Occupational stress negatively and indirectly affected job performance through moral intelligence (β =—0.560, t-value = 14.773, *p* < 0.01). Occupational stress also positively and indirectly impacted job performance through emotional intelligence (β = 0.098, t-value = 2.177, *p *< 0.01).

**Conclusions:**

Occupational stress slightly affects nurses’ job performance, and emotional and moral intelligence mediates the impact of occupational stress and improves nurses’ job performance. Low occupational stress improves nurses’ job performance, but too much occupational stress could harm nurses’ job performance. Healthcare administrators should work to help reduce nurses’ occupational stress and improve their job performance by adopting practical strategies to help nurses manage and control their stress. Holding stress reduction classes, eliminating the nursing shortage, reducing working hours, reducing workload, and providing financial and spiritual support to nurses would be recommended. It is also recommended to provide theoretical and practical emotional and moral intelligence-oriented courses for nursing students and hold training workshops for nurses to improve their emotional and moral intelligence.

## Background

Nursing care is crucial in improving patient satisfaction and safety, so paying attention to nurses’ Job performance has increased during the last decades [1]. Nurses’ Job performance depends on different factors such as job satisfaction, organizational commitment, and occupational stress [2]. The effect of occupational stress on job performance has been confirmed in several studies [3–5]. Occupational stress is defined as a person’s response to external-environmental stimuli. The negative impact of occupational stress on nurses’ Job performance has been shown in several studies [3, 6]. Due to the nature of nursing, nurses face countless problems that cause significant stress [7]. These problems include a shortage of human resources, heavy workload, tight work shifts, contradictions in the rhythm of life due to rotating shifts, exposure to human suffering and death, complex decision-making, moral dilemmas, etc. [8]. Statistics show that occupational stress is high among nurses in most countries [9–11]. In the meantime, the rate of occupational stress has been reported to be about 23.4–60% among Iranian nurses [12, 13]. Stress in the nursing work environment can cause many harmful consequences, including the inability to perform tasks correctly, low morale, no motivation to work, poor occupational performance, reduced productivity, and mental and physical weakness [14]. Occupational stress among nurses can be affected by various factors, especially moral and emotional intelligence [15, 16]. Mazzella Ebstein et al. reported a positive correlation between occupational stress and emotional intelligence among nurses working in national cancer institutes in the northeastern United States [17].

The term " emotional intelligence " was first described by Salovey and Mayer (1990) as "the ability to monitor one’s own and others’ emotions, distinguish between them, and use the information to guide one’s thoughts and actions" [18]. Emotional intelligence is a relatively stable individual ability, and a person can improve it through education and learning. It is also an important characteristic for proper nursing performance in different areas of nursing, including leadership, stress management, and caring functions [19]. Since nursing is considered a stressful profession, understanding how to manage stress is an important emotional intelligence skill [20]. Nursing care is provided in dynamic environments, and nurses with high emotional intelligence can manage chaotic environments, improve their thinking, make critical decisions, and use evidence in practice. In other words, emotions are established in human activities, behaviors, and characteristics as they can affect commitment to the responsivity of providing services for others and trust in oneself and others, all of which originate from moral intelligence [21]. Rajabi et al. showed that emotional and moral intelligence have a significant positive relationship in nursing students [22]. In another study, Barida et al. realized a positive association between high moral reasoning and high emotional intelligence. They indicated that these two issues could predict individuals’ moral behavior [23].

Lennick and Kiel (2011) defined moral intelligence as "the mental capacity to determine how human principles should be applied to individual values, goals, and actions" [24]. The main characteristics of moral intelligence consist of conscience, honesty, responsibility, compassion, forgiveness, continence, kindness, tolerance, justice, empathy, and respect, five of which emphasize "continence" [24, 25]. The presence of moral intelligence in nursing leads to "nursing professional development," "comprehensive care facilitation," and "organizational improvement" [26]. There has always been a possibility of conflict between individual values and ethical standards in clinical nursing [27]. A person with high moral intelligence not only does not think about him/herself but also determines appropriate and inappropriate actions in social relations, understands the feelings of others, empathizes with them, and takes responsibility for his/her actions [28]. Accordingly, moral intelligence is one of the components that can influence nurses’ performance, improve patient safety, and increase the quality of care [16]. Most studies investigated the effect of occupational stress on Job performance and reported contradictory results. However, the extent of the mediating effect of moral and emotional intelligence on nurses’ job performance is unclear. Thus, this study aimed to determine the effect of occupational stress on nurses’ Job performance and the mediating impact of moral and emotional intelligence.

## Conceptual framework and development of hypotheses

### Occupational stress and job performance

Several studies have examined the effect of occupational stress on nurses’ Job performance [3–5]. Some recent studies have shown a negative relationship between occupational stress and Job performance [4, 5, 29]. However, some previous studies reported the opposite results, and their findings indicated a positive and significant relationship between occupational stress and employees’ performance, so occupational stress encourages employees to work hard and improve their performance [5, 30, 31]. According to the model presented by Deng et al. (2019), occupational stress has a positive and significant relationship with Job performance among healthcare workers [3]. Therefore, the research team came to the following hypotheses:

- Hypothesis 1: Occupational stress has a positive effect on nurses’ performance.

### The mediating effect of emotional intelligence

Previous research showed that employees’ emotional intelligence significantly affects their performance [32, 33]. Zhu et al. (2015) showed that employees with high emotional intelligence had high engagement at work and would have better Job performance [34]. Ruble et al. (2022) indicated that pharmacists with high emotional intelligence had low occupational stress, increased mental health, and high professional integrity [33]. In the model presented by Rafiee et al. (2013), occupational stress positively and significantly affected emotional intelligence [35]. In contrast, studies have confirmed the negative impact of occupational stress on emotional intelligence [36, 37]. A study reported the positive mediating effect of emotional intelligence in the relationship between occupational variables and the Job performance [38]. Hence, emotional intelligence can improve nurses’ job performance by mediating the negative effect of occupational stress on their performance.

- Hypothesis 2: Occupational stress has a negative effect on nurses’ emotional intelligence.
- Hypothesis 3: Emotional intelligence has a positive effect on nurses’ job performance.
- *The main hypothesis*: Emotional intelligence mediates the effect of occupational stress on nurses’ job performance.

### The mediating effect of moral intelligence

Recent studies had contradictory results regarding the effect of moral intelligence on nurses’ performance. In Iran, Shakeri et al. (2021) reported no significant relationship between moral intelligence and the caring behaviors of pediatric nurses [39]. In contrast, Khosravani et al. (2020) revealed that moral intelligence improves nurses’ job performance by increasing organizational commitment and promoting the quality of patient care [40]. In the model developed by Rafiee et al. (2013), the positive effect of occupational stress on moral intelligence and the direct effect of moral intelligence on job performance were confirmed [35]. Considering the positive effect of moral intelligence on nurses’ occupational stress [16] and their job performance shown in recent studies [41, 42], the research team came to the following hypotheses:

- Hypothesis 4: Occupational stress has a positive effect on nurses’ moral intelligence.
- Hypothesis 5: Moral intelligence has a positive effect on nurses’ job performance.
- *The main hypothesis*: Moral intelligence mediates the effect of occupational stress on nurses’ job performance.

## Methods

### Research design and sampling

This descriptive cross-sectional study was conducted in medical centers affiliated with Urmia University of Medical Sciences (Imam Khomeini, Motahari, Taleghani, Kosar, and Seyed al-Shohada Hospitals) in 2022. The sample-to-item ratio was used to determine the sample size, and this ratio should not be less than 5:1 [43]. The questionnaires utilized for data collection were made up of 122 items. The minimum sample size was calculated to be 610. Regarding the possibility of 15% sample attrition, 700 nurses were considered the final sample size to cover the path analysis and mediation hypothesis. Participants were entered into the study using the quota sampling method.

First, the total population of nurses working in Urmia teaching hospitals was determined (*N* = 3985), and then the number of the requested sample size (*n* = 700) was divided by it.

$$\frac{\mathrm{n}}{\mathrm{N}}= \frac{700}{3985}=0.1756$$

**Fig. 1:**
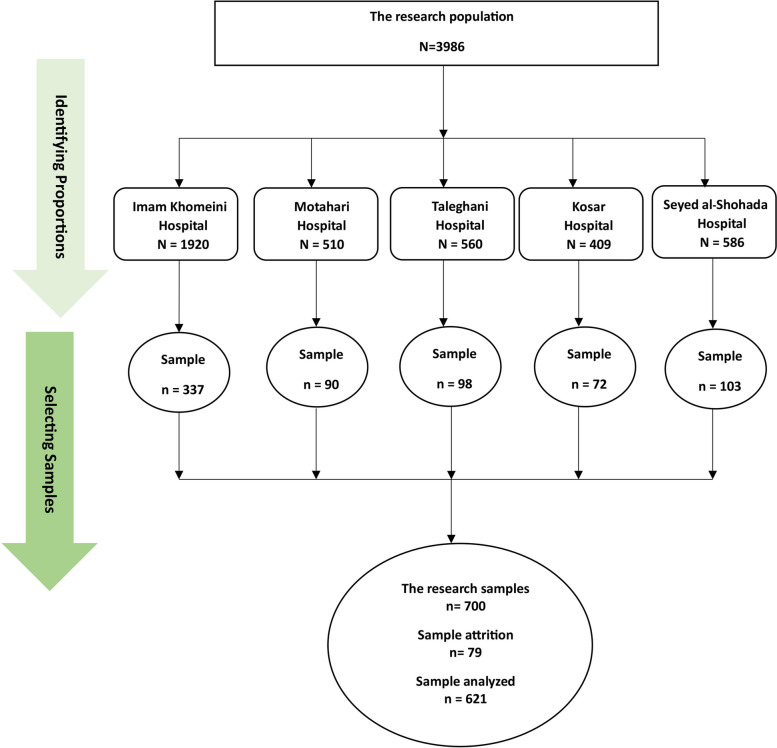
Study sampling flow diagram

Second, the obtained fraction (0.1756) was multiplied by the number of each stratum to determine how many nurses should be selected from each hospital (Fig. 1).

The inclusion criteria included working as a nurse in one of the Urmia teaching hospitals, having work experience of more than one year, and willingness to participate in the study. Incomplete questionnaires were excluded from the study.

### Procedure

After obtaining approval from the Research Ethics Committee and the Vice-Chancellor for Research & Technology, the lead researcher referred to the officials of the target hospitals. The researcher obtained the contact information of nurses from the nursing administration office. The participants were provided with the necessary explanations about the study objectives and how to complete the questionnaires. All participants assured that their participation was optional; they could withdraw anytime. They also assured of the confidentiality and anonymity of their data. If they wanted to participate in the study, written informed consent was sent to them using WhatsApp. The nurses signed the informed consent form and returned it to the researcher before entering the study. The questionnaires were sent to the nurses online. They should open the link and complete the questionnaire. They were requested to complete and return the questionnaire within 72 h. If nurses did not return the questionnaire within 48 h, they were asked to complete and return it for the second and third time. They were removed from the list if they did not complete and send the questionnaire after the third request. Out of 700 questionnaires, 621 questionnaires were completely filled in and returned. The data collection process lasted for three months.

#### Measures

Data were collected using demographics and four standard questionnaires described below:

I. The demographic questionnaire included age, gender, educational level, marital status, current occupational position and employment status, and the number of children.
II. Nursing Stress Scale (NSS)

The NSS is a 34-item questionnaire that Gray-Toft & Anderson developed in 1981. This questionnaire consists of 7 subscales, including patient mortality (7 items), conflict with doctors (5 items), insufficient preparation (3 items), problems with other nurses (6 items), lack of support (3 items), workload (6 items), and uncertainty about treatments (5 items). All items are scored on a 5-point Likert scale from "Never stressed = 1" to "Highly stressed = 4."To obtain the score for each subscale, the scores of all the items related to that subscale are added together. The overall score on the scale ranges from 34 to 136, and a higher score indicates higher occupational stress in a nurse [44–46]. Ghanei Gheshlagh et al. (2017) confirmed the reliability of this scale using Cronbach’s alpha coefficient of 0.96 [12]. Global Cronbach’s alpha of this scale was 0.92, which varied between 0.49 and 0.83 for each subscale [45]. Ten faculty members confirmed the scale’s validity in this study, and its Cronbach’s alpha was 0.89.

III. Paterson’s Job Performance Questionnaire (P-JPQ)

The P-JPQ is a 15-item tool developed by Patterson and Husband (1970). The scoring is based on a 4-point Likert scale from "Rarely = 0" to "Always = 3". The overall score of the questionnaire ranges between 0 and 45, where a high score indicates better job performance. Patterson and Husband (1970) confirmed its reliability using Cronbach’s alpha of 0.91 [47]. Hosseini et al. (2017) approved the face and content validity of the questionnaire as well as its reliability with Cronbach’s alpha coefficient of 0.84 [48]. In our study, the reliability of the questionnaire was confirmed in a pilot study with Cronbach’s alpha of 0.80.

IV. Siberia Schering’s Emotional Intelligence Standard Questionnaire (SS-EISQ)

The SS-EISQ is a 33-item tool with five subscales, including self-arousal (7 questions), self-awareness (8 questions), self-control (7 questions), sympathy/social awareness (6 questions), and social/communicational skills (5 questions). The items are scored on a 5-point Likert scale from "Never = 1" to "Always = 5". The range of overall scores is between 33 and 165 [49]. In Iran, this questionnaire was psychometrically evaluated by Mansouri in 2001, and its validity and reliability were confirmed. Cronbach’s alpha of the questionnaire was 0.84 [50]. In our study, the validity of the questionnaire was confirmed by ten faculty members, and its reliability was confirmed using the test–retest method with Cronbach’s alpha of 0.84.

V. Lennik and Keil’s Moral Intelligence Questionnaire (LK-MIQ)

The LK-MIQ is a 40-item tool developed by Lenik and Kiel in 2005. This questionnaire consists of four subscales with ten items each. The subscales include integrity, responsibility, compassion, and forgiveness. The scoring is based on a 5-point Likert scale from "Never = 1" to "Always = 5." The initial score ranges between 40–200, and the final score is calculated by dividing the initial score by two. The final score ranges between 20 and 100 [51]. In Iran, Majidi et al. (2018) approved the reliability of this questionnaire with Cronbach’s alpha coefficient of 0.88 [52]. In the present study, ten experts confirmed the face and content validity of the questionnaire. Furthermore, the reliability was confirmed in a pilot study using the test–retest method with Cronbach’s alpha of 0.91.

#### Data analysis

In this study, data analysis was conducted using IBM SPSS Statistics for Windows, version 23 (IBM Corp., Armonk, N.Y., USA) and SmartPLS version 2. Quantitative variables were reported using "mean ± standard deviation," and qualitative variables were reported using numbers (percentages) in the format of standard tables and graphs. The Fronell-Larcker criterion and the consistency method (Cronbach’s alpha coefficient) were used to check the discriminant validity and the model’s reliability, respectively. Path (β) and t coefficients were used to examine the conceptual model. A *p*-value of less than 0.05 was considered significant.

## Results

A total of 621 questionnaires were completed in this study, and the acceptance rate was about 88.7%. The mean age of the participants was 33.47 ± 8.477 years, and their mean work experience was 9.27 ± 7.94 years. Regarding marital status, 370 (59.6%) were married, and 251 (40.4%) were single. Regarding gender, 229 (36.9%) participants were male, and 392 (63.1%) were female. In terms of education, 514 (82.8%) had a bachelor’s degree, 92 (14.8%) had a master’s degree, and 15 (2.4%) had a Ph.D. In terms of employment status, 158 (25.4%) were passing the compulsory-service-program nurses; 37 (6%) were corporate nurses, 90 (14.5%) were contract nurses, 78 (12.6%) were temporary-to-permanent nurse employees; and 258 (41.5) %) were permanent nurse employees. Regarding the number of children, 125 (20.1%) had one child, and 167 (26.9%) had two or more children. In terms of occupational position, 524 (84.4%) were ward nurses, 33 (5.3%) were staff nurses, 28 (4.5%) were head nurses, 28 (4.5%) were supervisors, and 8 (1.3%) were matrons.

The results showed that Cronbach’s alpha was above 0.7 for all variables, indicating the model has good internal reliability. Based on the Fronell-Larcker criterion, the discriminant validity of the model is acceptable (Table 1).

**Table 1:** Fornell and Larker method (discriminant validity)

Variables	Emotional intelligence	Occupational stress	Moral intelligence	Job performance
**Emotional intelligence**	1			
**Occupational stress**	- 0.401	1		
**Moral intelligence**	- 0.317	0.148	1	
**Job performance**	0.121	0.085	- 0.548	1

### General conceptual model

**Fig. 2:**
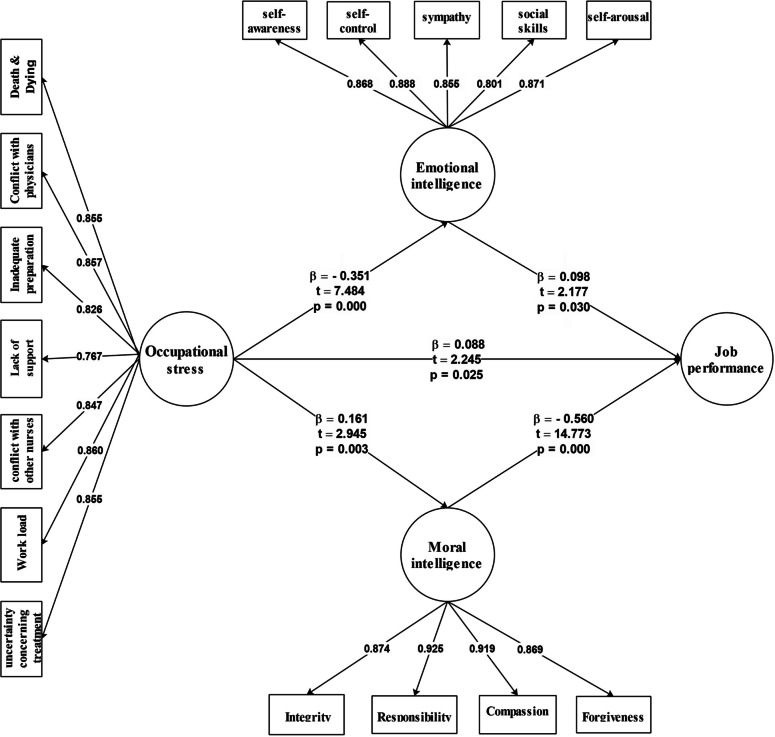
The direct model (*N* = 621)

**Fig. 3:**
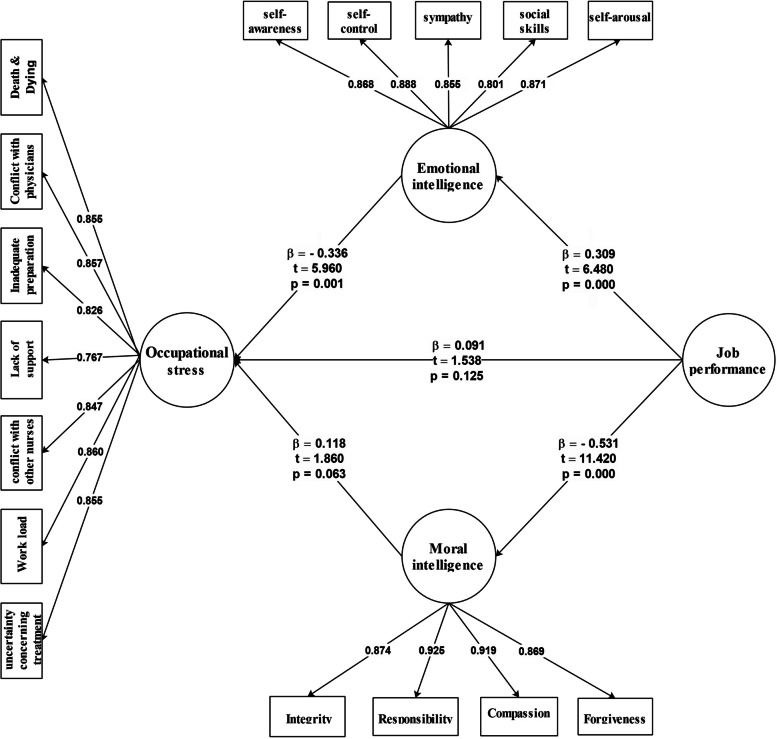
The indirect model (*N* = 621)

The conceptual model was examined after testing the research hypotheses. The results were extracted in path coefficients (ß) and t coefficients. The results extracted from the model analysis are shown in Figs. 2 and 3, and Table 2. Path coefficients indicated the direct effect of one construct on another construct. If the path coefficients are greater than 0.6 between the variables, the predictive effect of the latent variable is stronger than the dependent variable. The predictive impact is moderate when ß is between 0.3 and 0.6, and when it is less than 0.3, the predictive effect is considered weak. Based on the results in Fig. 2, occupational stress had a positive and minor impact on job performance (ß = 0.088) and moral intelligence (ß = 0.161); moral intelligence had a negative and medium effect on job performance (ß = -0.560); occupational stress had a negative and medium impact on emotional intelligence (ß = -0.351); and emotional intelligence had a positive and minor effect on job performance (ß = 0.098) (Fig. 2).

**Table 2:** Results of structural equation analysis for the general conceptual model

**Path**	*β*	*t*	*Sig*	*Result*
**P1:** Occupational stress → Job performance	0.088	2.245	= 0.025	Confirmed
**P2:** Occupational stress → Moral intelligence	0.161	2.945	= 0.003	Confirmed
**P3:** Moral intelligence → Job performance	- 0.560	14.773	= 0.000	Rejected
**P4:** Occupational stress → Emotional intelligence	- 0.351	7.484	= 0.000	Confirmed
**P5:** Emotional intelligence → Job performance	0.098	2.177	= 0.030	Confirmed

Figure 2 showed that the t-statistics for examining the relationship between research variables were above 1.96. Accordingly, with at least 95% confidence, the relationships between variables significantly affected each other (Fig. 2).

The standard path coefficients and *t*-statistics were presented at the 95% confidence interval based on the graphical findings for the general conceptual model (Table 1). Regarding the existence of two mediating variables in this study, the indirect relationships between the variables were examined using the macro bootstrap method. The results of the multiple mediation test of indirect relationships for the entire sample are shown in Table 2.

The bootstrap test results showed that the upper and lower bounds of the indirect relationship between occupational stress and job performance with the mediating effect of moral intelligence did not contain zero, meaning that this indirect path was significant in the entire sample (the mediating effect was confirmed). Moreover, the upper and lower bounds of the indirect relationship between occupational stress and job performance with the mediating impact of emotional intelligence did not contain zero, meaning that this indirect path was significant in the entire sample (the mediating effect was confirmed) (Table 3).

**Table 3:** Indirect effects and bootstrapping results with all paths (multiple mediation analysis)

**Path**	*Indirect effect*	*Bootstrap standard errors*	*95% Bootstrapped Confidence Interval*	
			*Lower*	*Upper*
Occupational stress to Job performance through **Moral intelligence**	- 0.0536	0.0118	- 0.0775	- 0.0311
Occupational stress to Job performance through **Emotional intelligence**	- 0.0111	0.0058	-0.0230	- 0.0003

Sixteen observed variables and four latent variables, namely occupational stress, job performance, emotional intelligence, and moral intelligence, were included in the hypothetical model. The measurement model had a good fit (*Χ*^*2*^ = 16535.28, *df* = 7253, *P* = 0.0001, *RMSEA* = 0.052; *NNFI* = 0.95; *PNFI* = 0.9; *IFI* = 0.95; *GFI* = 0.91, *NFI* = 0.92, *CFI* = 0.95; *RFI* = 0.92). All factor loadings for latent variable indicators were also significant (*P* < 0.001), which means that all latent variable indicators represent them well.

## Reverse model

According to the output of the reverse model diagram, the effect of job performance on occupational stress was positive and small (ß = 0.091); the impact of job performance on moral intelligence was negative and medium (ß = -0.531); the effect of moral intelligence on occupational stress was positive and small (ß = 0.118); the impact of job performance on emotional intelligence was positive and medium (ß = 0.309); and the effect of emotional intelligence on occupational stress was negative and medium (ß = -0.336). The results showed that some of the path coefficients between the latent variables were not statistically significant, and the fit indices of the reverse model were not satisfactory (P (RMSEA) > 0.05). Therefore, the reverse model was not acceptable (Fig. 3).

The relationship between demographic characteristics and the main study variables is presented in Table 4.

**Table 4:** Linear regression model between moral intelligence, emotional intelligence, occupational stress and job performance with demographic variables

Variables	Moral intelligence		Emotional intelligence		Occupational Stress		Job performance	
	B	*P*-Value	B	*P*-Value	B	*P*-Value	B	*P*-Value
**Constant**	97.445	0.001	108.381	0.001	81.298	0.001	32.655	0.001
**Age**	-0.309	0.228	-0.136	0.37	-0.093	0.664	0.169	0.062
**Gender**	0.73	0.677	0.503	0.627	-7.24	0.001	-1.475	0.017
**Marital status**	0.678	0.718	0.325	0.77	-0.704	0.654	0.071	0.914
**Educational level**	-0.029	0.988	2.658	0.014	-0.539	0.724	-0.801	0.216
**Number of children**	2.568	0.055	-0.239	0.762	0.952	0.393	-1.258	0.008
**Current occupational position**	-0.26	0.333	0.318	0.045	0.119	0.595	0.106	0.265
**Employment status**	0.004	0.987	-0.408	0.011	0.551	0.015	-0.25	0.009

## Discussion

This study was conducted to determine the effect of occupational stress on nurses’ Job performance and the mediating impact of moral and emotional intelligence.

In this study, nurses’ occupational stress had a positive and minor effect on their job performance. In line with our results, Deng et al. (2019) showed that limited occupational stress can improve job performance among health workers [3]; the possible explanation is that low occupational stress can increase concentration among nurses, improving their performance. Another possible reason for the improvement in nurses’ performance in this study is that most nurses have nearly nine years of work experience, which can be effective in controlling their stress and improving their performance. Some studies reported a significant negative relationship between work experience and occupational stress [17, 53]. It seems that more experienced nurses had better adaptation to the hardship of working environments and had a greater capability to deal with workplace tension, so they had less occupational stress.

In contrast with our results, some previous studies reported the adverse effects of occupational stress on job performance [4, 5, 29] and concluded that higher stress could decrease concentration and job performance [3, 54]. Nurses face many challenges, including workforce shortage, high workload, lack of professional independence, and lack of management support, so they experience high occupational stress [12, 55]. High stress leads to sleep disorders, depression, loss of concentration, and a significant decrease in job performance [10–12].

The results indicated that emotional intelligence indirectly mediates the effect of occupational stress on nurse’s job performance. Moreover, emotional intelligence improves nurses’ job performance by reducing their occupational stress. Cano and Sams (2009) showed that employees with high emotional intelligence perform better emotional behaviors in the work environment and experience less occupational stress [56], causing them to work more effectively and have better performance [32, 57]. Based on Joseph and Newman’s cascading model, emotional intelligence begins with receiving emotion, and regulating and understanding emotions leads to high job performance. People with healthy emotion regulation can choose more appropriate approaches to job demands, which helps them drain fewer resources and maintain their job performance at a high level [58, 59]. Employees with high levels of emotional intelligence are more optimistic and have the power to change and manage stressful situations in their work environment without being affected by surrounding factors. These people also actively act when facing occupational stress [60].

In contrast with our study’s findings, Rafiee et al. (2013) revealed that occupational stress positively affected emotional intelligence. They also showed that emotional intelligence had no significant effect on job performance and did not mediate the impact of occupational stress [35]. The possible reason for this discrepancy between the results is that the sample size in their study was much smaller than ours and could affect the findings.

The results also showed that moral intelligence indirectly mediates the effect of occupational stress on nurses’ job performance. Namely, moral intelligence improves nurses’ job performance by reducing their occupational stress. Similar to our study result, Rafiee et al. (2013) showed that moral intelligence acts as a mediator between occupational stress and job performance, enhancing employee performance [35]. As an inherent support factor, moral intelligence can improve nurses’ psychological safety by strengthening positive psychological factors. Decent moral intelligence can also reduce occupational stress among employees and improve their performance [16]. Moreover, it was shown that moral intelligence enhances employee job satisfaction and performance by improving their mental health [61]. An individual with high moral intelligence better understands stressful situations, so he/she can have appropriate moral behavior in such situations [62].

Our results indicated that moral intelligence had no significant relationship with demographic characteristics. Emotional intelligence was significantly related to education level, employment status, and nurse position, which was in line with the Ebrahimi et al. study findings [63]. Similar to Yousefi et al.’s results, occupation stress had a significant relationship only with gender and employment status [64]. Job performance was significantly related to gender, number of children, and employment status. In Ogunleye’s study, job performance had a significant relationship with employment status. In contrast, no significant effect of gender on job performance was found in their research, although the interactive effect of gender and employment status was related to the personality characteristics and the participant’s skills [65].

### Study strengths & limitations

This study is the first to examine the mediating effect of emotional and moral intelligence on nurses’ job performance. However, since emotional and moral intelligence are strongly influenced by people’s culture and spiritual background, researchers should act cautiously in generalizing the results. Another limitation was the existence of confounders, such as the type of shift work and personal characteristics, which could affect the main variables, namely occupational stress and job performance. Therefore, it is recommended to identify confounding factors and control their effects in future studies.

## Conclusion

Occupational stress has a positive and minor effect on nurses’ job performance. Also, emotional and moral intelligence mediates occupational stress’s effect and improve nurses’ job performance. Low occupational stress improves nurses’ job performance, but too much occupational stress could harm nurses’ job performance. Healthcare administrators should work to help reduce nurses’ occupational stress and improve their job performance by adopting practical strategies to help nurses manage and control their stress. The research team recommends that nursing managers take a big step in reducing nurses’ occupational stress by holding stress reduction classes, eliminating the shortage of nurses, reducing working hours, reducing workload, and providing financial and spiritual support to nurses. In addition, it is recommended to provide theoretical and practical emotional and moral intelligence-oriented courses for nursing students and hold training workshops for nurses to improve their emotional and moral intelligence.

## Data Availability

The datasets generated during and/or analyzed during the current study are available from the corresponding author on reasonable request.

## Abbreviations

NSS: Nursing Stress Scale
P-JPQ: Paterson’s Job Performance Questionnaire
SS-EISQ: Siberia Schering’s Emotional Intelligence Standard Questionnaire
LK-MIQ: Lennik and Keil’s Moral Intelligence Questionnaire
SPSS: Statistical Package for the Social Science
RMSEA: The Root Mean Square Error of Approximation
NNFI: Non-Normed Fit Index
PNFI: Parsimony Normed Fit Index
IFI: Incremental Fit Index
GFI: Goodness of Fit Index
NFI: Normed Fit Index
CFI: Comparative Fit Index
RFI: Relative Fit Index
